# Set-membership estimations for the evolution of infectious diseases in heterogeneous populations

**DOI:** 10.1007/s00285-016-1050-0

**Published:** 2016-09-07

**Authors:** Tsvetomir Tsachev, Vladimir M. Veliov, Andreas Widder

**Affiliations:** 1 Institute of Mathematics and Informatics, Bulgarian Academy of Sciences, Acad. G. Bonchev Str., Block 8, 1113 Sofia, Bulgaria; 2 ORCOS, Institute of Statistics and Mathematical Methods in Economics, Vienna University of Technology, Wiedner Hauptstraße 8/E105-4, 1040 Vienna, Austria

**Keywords:** Epidemic models, Uncertain distributed systems, Set-membership estimation, Heterogeneous population models, SI, SIR disease models, 92D30, 49J53, 35F25, 35Q92

## Abstract

The paper presents an approach for set-membership estimation of the state of a heterogeneous population in which an infectious disease is spreading. The population state may consist of susceptible, infected, recovered, etc. groups, where the individuals are heterogeneous with respect to traits, relevant to the particular disease. Set-membership estimations in this context are reasonable, since only vague information about the distribution of the population along the space of heterogeneity is available in practice. The presented approach comprises adapted versions of methods which are known in estimation and control theory, and involve solving parametrized families of optimization problems. Since the models of disease spreading in heterogeneous populations involve distributed systems (with non-local dynamics and endogenous boundary conditions), these problems are non-standard. The paper develops the needed theoretical instruments and a solution scheme. SI and SIR models of epidemic diseases are considered as case studies and the results reveal qualitative properties that may be of interest.

## Introduction

The role of heterogeneity of a population for the evolution of infectious diseases is well recognized in the existing literature, see e.g. Diekmann et al. (1990, (2012), Coutinho et al. (1999). Various kinds of models have been developed to take into account heterogeneity with respect to immune system, contact rates and other traits, including cellular automata (Schneckenreither et al. 2006), random networks (Miller 2007; Volz 2008), distributed integro-differential systems (Novozhilov 2008, 2012; Diekmann et al. 1990; Veliov 2005), etc. For a more comprehensive bibliography see the recent paper (Hickson and Roberts 2014). Theoretical results about the influence of heterogeneity on the basic reproduction number in such models are available (e.g. Katriel 2012; Margheri et al. 2015), as well as results about intervention strategies, e.g. vaccination in heterogeneous populations (Rodrigues et al. 2009; Katriel 2012). A substantial limitation for utilization of most of these models is that they require detailed information about the distribution of the population along the numerical values of the traits, that is, about the *h*-state (heterogeneous state) of the individuals in the population (Diekmann et al. 2012). Such detailed information is usually not available. The available information is vague and even reliable statistical characteristics are often not known. One way to overcome this difficulty is to pass to aggregated models that require less information. This approach is stressed in Diekmann et al. (2012) and we mention the papers (Dushoff 1999; Karev 2005, 2010; Veliov 2005; Novozhilov 2008, 2012) developing aggregation techniques for certain special classes of heterogeneous models defined by integro-differential systems.

In the present paper we employ an alternative approach, in which the distribution of the population among the *h*-states is uncertain, but set-membership information is available (possibly together with certain aggregated data). The set-membership information may be given in the form of lower and upper bounds for the number of susceptible, infected, recovered, etc. individuals at each *h*-state. The aggregated information is typically about the total number of susceptible, infected, etc. individuals at the initial time. This information is used to obtain set-membership estimations (shortly: set-estimates) for the evolution of the disease. The set estimations at a given time *t* contain all aggregated states (total number of susceptible, infected, etc. individuals) that are consistent with the available initial information and the model describing the dynamics of the population system. The set-estimation approach is well known and widely used (see e.g. Bertsekas 1995; Milanese and Vicino 1996; Kurzhanski and Varaiya 2011), but in the present epidemiological context there are important points that had to be developed.

The investigation is carried out for a rather general model of a heterogeneous multi-group population, which consists of a distributed first order differential system complemented with integral relations. This model covers heterogeneous versions of SI, SIR, and many other standard epidemiological models. At this level of generality we present our set-estimation approach. In the set-estimation theory for evolutionary systems one can distinguish two different groups of methods. In the first, set-estimations of Markovian type are sought, where the set-estimation at a given time *t* determines the future set-estimations (the minimal set-estimation has this property). The advantage is, that it is sometimes possible to obtain infinitesimal (even differential) equations for the evolution of Markovian set-estimations in a prescribed family of sets (polyhedrons, ellipsoids, etc., see Kurzhanski and Varaiya 2011). The drawback is, that such estimations are usually too “pessimistic” , that is, too large, compared with the minimal set-estimation. Our approach belongs to the second group: at each time the set-estimation is obtained independently of the previously obtained estimations. Technically, finding such estimations (even minimal ones) can be done by solving families of auxiliary dynamic optimization problems. In our case these optimization problems are non-standard, because they involve constraints in the form of first order distributed differential systems and integral relations. Therefore, the first main goal of the paper is to present a technique for solving such optimization problems.

The second goal of the paper is to show that the set-estimation technique may give useful information about the spread of infectious diseases under uncertainty of data (we focus on uncertainty of the *h*-state-distribution of the initial population). In many cases the population has certain dissipativity property that makes the set-estimations not much expanding, even shrinking to a point or to a reasonably small set, when the time progresses. Thanks to this, one can perform various kinds of comparative analysis. For example, we investigate the effect of various scenarios of interventions (vaccination or prevention programs) applied prior to the outburst of the disease.

We mention that our previous work (Veliov and Widder 2015) allows to determine the exact asymptotics of the aggregated states of a class of heterogeneous SI-models, depending on the initial *h*-state-distribution of the population. This allows to obtain a set-estimation for the asymptotic state of the disease for this particular SI-model in an alternative way. The comparison with the results obtained by the general approach in the present paper, which turns out to be the same, serves as a verification test.

The plan of the paper is as follows. Section 2 explains the aim of this paper in terms of a simple SI model used later as benchmark. The general model, the assumptions, and the formulation of the set-estimation problem are given in Sect. 3. In Sect. 4 we present the set-membership technique and some technicalities needed to adapt it to the present framework. Section 5 is devoted to numerical analysis of certain SI and SIR heterogeneous models by the set-estimation techniques. Some conclusions and lines of future research are presented in Sect. 6. Some technical proofs are given in the Appendices 1 and 2.

## A benchmark SI model

To present our main motivation, we introduce below a particular case of the problem we investigate in this paper, which involves a heterogeneous version of the known SI model in mathematical epidemiology. The whole population is divided into two groups—susceptible individuals and infected individuals. The individuals are heterogeneous, in the sense that a scalar $\omega \in \varOmega \subset \mathbf{R}$ is associated with each individual, indicating specific traits relevant to the particular disease, e.g. the intensity of risky contacts, the state of the immune system, etc. The parameter $\omega$ is called heterogeneous state (shortly *h*-state) of the individual, see e.g. Coutinho et al. (1999), Diekmann et al. (1990) or textbooks such as Diekmann et al. (2012).

The following model is a particular case of the one in Veliov (2005):

1 $$\begin{aligned} \dot{S}(t,\omega )= & {} -\sigma (\omega ) p(\omega )\frac{J(t)}{K(t)+J(t)}S(t,\omega )+\kappa S(t,\omega ), \quad S(0,\omega )=u_1(\omega ),\nonumber \\ \dot{I}(t,\omega )= & {} \sigma (\omega ) p(\omega )\frac{J(t)}{K(t)+J(t)}S(t,\omega ) -\gamma I(t,\omega ),\quad I(0, \omega )=u_2(\omega ),\nonumber \\ K(t)= & {} \int _\varOmega p(\omega ) S(t,\omega ) \text{ d }\omega ,\nonumber \\ J(t)= & {} \int _\varOmega q(\omega ) I(t,\omega ) \text{ d }\omega . \end{aligned}$$

Here $S(t,\omega )$ and $I(t,\omega )$ represent the size of the susceptible and of the infected population with *h*-state $\omega$ at time *t*, respectively. The parameter $\kappa$ is the net population growth rate of the susceptible population, $\gamma$ is the net mortality rate of the infected population, $\sigma (\omega )$ is the susceptibility, meaning the probability that a risky interaction between a susceptible and an infected individual results in infection of the susceptible individual (it may incorporate also the immune status of the susceptible individual), and $p(\omega )$ and $q(\omega )$ denote the participation rate of susceptible/infected individuals of *h*-state $\omega$ in risky interactions (i.e. the contact rate). The aggregated state variables *K*(*t*) and *J*(*t*) represent the total amount of susceptible/infected individuals, weighted with their respective risky behaviour, while $J(t)/(K(t) + J(t))$ is the weighted prevalence of the disease at time *t* (see e.g Veliov 2005; Veliov and Widder 2015 for more detailed explanations). At the initial time $t=0$, the distribution of the initial susceptible and infected sub-populations along the *h*-states, $\omega \in \varOmega$, is given by the functions $u_1(\omega )$ and $u_2(\omega )$, respectively.

In fact, the main quantities of practical interest are the total size of the susceptible and infected populations:

2 $$\begin{aligned} S(t) := \int _\varOmega S(t,\omega ) \text{ d }\omega \quad \text{ and } \quad I(t) := \int _\varOmega I(t,\omega ) \text{ d }\omega . \end{aligned}$$

Solving system (1) is not problematic, provided that all data involved are known. However, in reality the information about the distribution of individuals along the heterogeneous space $\varOmega$ is vague. That is, the functions $u_1$ and $u_2$ are not precisely known. A relatively reliable information about these functions is provided by the aggregated values

3 $$\begin{aligned} \int _\varOmega u_1(\omega ) \text{ d }\omega = S(0)\quad \text{ and } \quad \int _\varOmega u_2(\omega ) \text{ d }\omega = I(0), \end{aligned}$$

since measurements of *S*(0) and *I*(0) are feasible. Statistical information for higher integral moments of $u_1$ and $u_2$ (in the form of equalities or inequalities) may also be available, and its incorporation in our subsequent considerations is a matter of technical work that we avoid for more transparency. Additional information about $u_1$ and $u_2$ may be given in terms of bounds:

4 $$\begin{aligned} u_i(\omega ) \in [\varphi _0^{i}(\omega ), \varphi _1^{i}(\omega )], \quad \omega \in \varOmega , \quad i = 1,\,2. \end{aligned}$$

Any pair of measurable functions $(u_1,u_2)$ satisfying (3) and (4) (that is, consistent with the available information) will be viewed as possible (sometimes called admissible) realizations of the uncertainty for the *h*-distribution of the initial population.

Due to the uncertainty of the initial data $(u_1,u_2)$, the issue of obtaining a set-membership estimation, $\mathcal{E}(t)$, of the aggregated state (*S*(*t*), *I*(*t*)) does naturally arise. This means that sets $\mathcal{E}(t), \; t \ge 0$, have to be determined, such that

5 $$\begin{aligned} (S(t),I(t)) \in \mathcal{E}(t), \quad t \ge 0, \end{aligned}$$

whatever the admissible initial functions $(u_1, u_2)$ are, where (*S*(*t*), *I*(*t*)) is the corresponding solution of system (1) enhanced with (2).

The main goal of this paper is to present a computationally implementable approach for obtaining set-membership estimations as in (5). Such an approach is developed in the next section for a general system with a structure similar to (1), (2).

## Formulation of the problem and preliminaries

Having in mind the set-membership estimation problem in the previous section, below we formulate a more general problem that covers heterogeneous versions of a variety of models in mathematical epidemiology and in other areas.

Let [0, *T*] be a given time-interval and let $\varOmega$ be a compact interval in which the parameter of heterogeneity, $\omega$, takes values. Denote $D = [0,T] \times \varOmega$. State variables in the model below are functions

$$\begin{aligned} x : D \rightarrow \mathbf{R}^m \quad \text{ and } \quad y : [0,T] \rightarrow \mathbf{R}^n. \end{aligned}$$

The dynamics is given by the equations

6 $$\begin{aligned} \dot{x}(t,\omega )= & {} f(t,\omega ,x(t,\omega ),y(t)), \quad \; (t,\omega ) \in D, \end{aligned}$$

7 $$\begin{aligned} x(0,\omega )= & {} u(\omega ), \quad \qquad \; \omega \in \varOmega , \end{aligned}$$

8 $$\begin{aligned} y(t)= & {} \int _{\varOmega } g(t,\omega ,x(t,\omega )) \text{ d }\omega , \quad t \in [0,T], \end{aligned}$$

where

$$\begin{aligned} f : D \times \mathbf{R}^m \times \mathbf{R}^n \rightarrow \mathbf{R}^m \quad \text{ and } \quad g : D \times \mathbf{R}^m \rightarrow \mathbf{R}^n \end{aligned}$$

are given functions and the upper “dot” means differentiation with respect to *t*, so that $\dot{x}(t,\omega ) := \partial x(t,\omega )/\partial t$. The initial data $u: \varOmega \rightarrow \mathbf{R}^n$ is uncertain, and the available information about it is given by the following constraints:

9 $$\begin{aligned}&u(\omega ) \in [\varphi _0(\omega ), \varphi _1(\omega )], \quad \omega \in \varOmega , \end{aligned}$$

10 $$\begin{aligned}&\quad \int _\varOmega u(\omega ) \text{ d }\omega = c. \end{aligned}$$

The inclusion in (9) is understood component-wise: $u_i(\omega ) \in [\varphi _0^{i}(\omega ),\varphi _1^{i}(\omega )]$, where $u = (u_1, \ldots , u_m)$, $\varphi _j = (\varphi _j^{1}, \ldots , \varphi _j^{m})$, $j = 0,\,1$; the vector $c \in \mathbf{R}^m$ and the functions $\varphi _0$ and $\varphi _1$ are given.

We consider every function from the set

$$\begin{aligned} \mathcal{U}:= \left\{ u \in L_{\infty }^m (\varOmega ) : u(\omega ) \in [\varphi _0(\omega ), \varphi _1(\omega )] \;\; \text{ for } \text{ a.e. } \omega \in \varOmega , \;\int _\varOmega u(\omega ) \text{ d }\omega = c \right\} . \end{aligned}$$

as an admissible (possible) realization of the uncertain function *u*.

Before formulating the estimation problem in the spirit of the previous section, we give the necessary assumptions and clarify the meaning of solution of the above model.

*Assumptions:*

(i) The function *f* is measurable in $(t,\omega )$, *g* is continuous in *t* and measurable in $\omega$, both are locally essentially bounded, differentiable in (*x*, *y*) with locally Lipschitz partial derivatives, uniformly with respect to $(t,\omega ) \in D$;
(ii) the functions $\varphi _0, \, \varphi _1 : \varOmega \rightarrow \mathbf{R}^m$ are continuous and satisfy the inequalities $\varphi _0(\omega ) \le \varphi _1(\omega )$ and $\int _\varOmega \varphi _0(\omega ) \text{ d }\omega< c < \int _\varOmega \varphi _1(\omega ) \text{ d }\omega$;
(iii) for every $u \in \mathcal{U}$, a unique solution (*x*[*u*], *y*[*u*]) of (6)–(8) does exist on the whole interval [0, *T*]. Moreover, (*x*, *y*) is uniformly bounded in $L_\infty (D) \times L_\infty ([0,T])$ when $u \in \mathcal{U}$.

By definition, solution of (6)–(8) is any pair of measurable and bounded functions $(x(\cdot ,\cdot ),y(\cdot ))$ on *D* and [0, *T*] respectively, such that for a.e. $\omega \in \varOmega$ the equation

11 $$\begin{aligned} x(t,\omega ) = u(\omega ) + \int _0^t f(s,\omega ,x(s,\omega ),y(s)) \text{ d }s \end{aligned}$$

holds on [0, *T*] and (8) holds for a.e. $t \in [0,T]$.

Notice that according to Assumption (i) $x(\cdot , \omega )$ is (uniformly) Lipschitz continuous for a.e. $\omega$ and *y* is continuous.

Existence and uniqueness of the solution (*x*[*u*], *y*[*u*]) on [0, *T*] for every $u \in \mathcal{U}$ is assumed in (iii) above in order to make the exposition less technical. In fact, the existence can be proved by a fixed point argument similarly as in Veliov (2008, Theorem 1) under an additional growth condition, which is not necessary in the rest of this paper. The uniqueness is standard due to the Lipschitz continuity in Assumption (i).

Denote

$$\begin{aligned} \mathcal{R}(t) := \left\{ y[u](t) : \,u \in \mathcal{U}\right\} , \quad t \in [0,T]. \end{aligned}$$

That is, $\mathcal{R}(t)$ the set of all aggregated states *y* that result from some admissible realization of the uncertainty, $u \in \mathcal{U}$. In this sense, $\mathcal{R}(t)$ is the exact (meaning *minimal*) set-membership estimation of the aggregated state at time *t*. In the next section we present a method of obtaining estimates

$$\begin{aligned} \mathcal{E}(t) \supset \mathcal{R}(t), \quad t \in [0,T]. \end{aligned}$$

Even more, the method allows to obtain outer approximations of arbitrary accuracy of the convex hull $\text{ co }\,\mathcal{R}(t)$.

Sometimes not all components of *y* are of interest (being included just to complete the model). Therefore, for a given subspace $L \subset \mathbf{R}^n$ we will obtain estimations of the projections of *y*(*t*) on *L*:

12 $$\begin{aligned} \mathcal{E}_L(t) \supset \text{ pr }_L (\text{ co }\,\mathcal{R}(t)), \end{aligned}$$

where $\text{ pr }_L$ is the projection operator on *L*.

In the epidemiological problems which serve as prototypes for the above problem (cf. Dushoff 1999; Hickson and Roberts 2014; Veliov 2005; Veliov and Widder 2015), the dimension *m* may equal 2 (in SI and SIS models), 3 (in SIR models), etc. The aggregated state *y* has usually a higher dimension than *x*. In the benchmark model (1), (2) considered in Sect. 2 we have $m=2$ and $n = 4$: $x(t,\omega ) = (S(t,\omega ), I(t,\omega ))$, $y(t) = (K(t),J(t),S(t),I(t))$. However, estimating the pair (*S*(*t*), *I*(*t*)) is of primal interest, thus $L := (0,0,\mathbf{R},\mathbf{R})$.

## The set-membership estimation

In this section we focus on the approximation of the exact set-membership estimation $\mathcal{R}(t)$. The procedure described in the first subsection is well known in control theory, while the second subsection is devoted to the main technical tool which is specific for the model presented in the previous section. The numerical scheme is briefly described in the third subsection.

### The approach

Let us fix a time $\tau \in (0,T]$ and a unit vector $l \in \mathbf{R}^n$. Consider the optimization problem

13 $$\begin{aligned} \sup _{u \in \mathcal{U}} \langle l, y(\tau ) \rangle = \sup _{y \in \mathcal{R}(\tau )} \langle l, y \rangle , \end{aligned}$$

subject to the constraints (6)–(10). (Here and below $\langle \cdot , \cdot \rangle$ denotes the scalar product.) Let $y_l = y[u_l](\tau )$ be an $\varepsilon$-solution, in the sense that

$$\begin{aligned} \langle l, y_l \rangle \ge \sup _{y \in \mathcal{R}(\tau )} \langle l, y \rangle - \varepsilon . \end{aligned}$$

If we find $y_{l_i}$ for a number of unit vectors $\{l_1, \ldots , l_k\} =: \varLambda$, then we have that

$$\begin{aligned} Y_\varLambda (\tau ):= & {} \{y_{l_1}, \ldots , y_{l_k}\} \subset \mathcal{R}(\tau ) \\&\subset \{ y \in \mathbf{R}^n : \,\langle l_i, y - y_{l_i} \rangle \le \varepsilon , \; i = 1, \ldots , k \} =: \mathcal{E}_\varLambda (\tau ). \end{aligned}$$

Thus, $\mathcal{E}_\varLambda (\tau )$ is a set-membership estimation of $y(\tau )$. It is an easy exercise to show that the Hausdorff distance $H(\text{ co }\,Y_\varLambda (\tau ), \mathcal{E}_\varLambda (\tau ))$ decreases with $\varepsilon$ and converges to zero if the set $\varLambda$ is a $\delta$-net on the unit sphere and $\delta$ and $\varepsilon$ converge to zero. Thus, we can obtain inner and outer approximations of any accuracy to the convex hull of the exact set-membership estimation $\mathcal{R}(\tau )$.

If only a set-membership estimation on a subspace $L \subset \mathbf{R}^n$ is needed (see (12)), then it is enough to take collections of unit vectors $\varLambda$ belonging to the space *L* (which makes problems of high dimension tractable, provided that the dimension of *L* is low—1, 2 or 3).

The approach described above requires multiple solving of problem (13), (6)–(10). This is not an easy task, since we deal with a distributed system with non-local dynamics (due to the presence of the aggregated states *y*) and constraints on the variable *u*. We employ a gradient projection method in the space $L_\infty (\varOmega )$ for the variable $u \in \mathcal{U}$. This means that the objective function in (13) is considered as a functional, *J*(*u*) of $u \in \mathcal{U}\subset L_\infty (\varOmega )$ with $y(\tau )$ viewed as a function of *u*: $y(\tau ) = y[u](\tau )$. The functional

14 $$\begin{aligned} J(u) = \langle l, y[u](\tau ) \rangle \end{aligned}$$

has to be maximized on the set $\mathcal{U}$. Then a standard gradient projection method can be implemented—for more details see Sect. 4.3 below.

However, there is an auxiliary problem that arises: to determine the gradient (meaning the Fréchet derivative) of *J*. This problem will be addressed in the next subsection.

### The gradient in problem (13)

Let $u \in \mathcal{U}$ and let (*x*, *y*) be the corresponding solution of system (6)–(8) on $[0,\tau ] \times \varOmega$, where $\tau \in (0,T]$ is the number fixed in the previous subsection. We shall obtain a representation of the Fréchet derivative of the functional *J* in (14) in the space $L_\infty$.

Let

$$\begin{aligned} \lambda : D \mapsto \mathbf{R}^m \quad \text{ and } \quad \nu : [0,\tau ] \mapsto \mathbf{R}^n \end{aligned}$$

be a measurable and bounded solution on $[0,\tau ] \times \varOmega$ of the system

15 $$\begin{aligned} -\dot{\lambda }(t,\omega )= & {} (f'_x(t,\omega ,x(t,\omega ),y(t)))^\top \lambda (t,\omega ) + (g'_x(t,\omega ,x(t,\omega )))^\top \nu (t), \end{aligned}$$

16 $$\begin{aligned} \lambda (\tau ,\omega )= & {} - (g'_x(\tau ,\omega ,x(\tau ,\omega )))^\top \,l, \end{aligned}$$

17 $$\begin{aligned} \nu (t)= & {} \int _\varOmega (f'_y(t,\omega ,x(t,\omega ),y(t)))^\top \lambda (t,\omega ) \text{ d }\omega . \end{aligned}$$

Here the superscript $\top$ means transposition, and the meaning of solution is similar to that of the initial-value problem (6)–(8).

#### Lemma 1

For every $u \in \mathcal{U}$ and corresponding solution (*x*, *y*) of system (6)–(8) a unique solution $(\lambda ,\nu )$ of system (15)–(17) does exist. Moreover, $\Vert \lambda \Vert _{L_\infty (D)} + \Vert \nu \Vert _{L_\infty (0,\tau )} \le {\bar{c}}$, where the constant ${\bar{c}}$ can be chosen independent of $u \in \mathcal{U}$.

The proof is similar to that of Proposition 1 in Veliov (2008). Therefore, we only sketch it. The solution of the linear equation (15) (with $\nu$ regarded as given) can be represented by the Cauchy formula, involving the fundamental matrix solution, which of course, depends on $\omega$. After inserting the resulting expression in (17) and changing the order of integration, we obtain a Volterra integral equation of the second kind for $\nu$, which has a unique solution. The uniform boundedness follows from the same property of (*x*, *y*) (see Assumption (iii)).

#### Proposition 1

The functional $J : L_{_{\infty }}(\varOmega ) \longrightarrow \mathbf{R}$ is Fréchet differentiable and its derivative at *u* has a representation in $L_{_{\infty }}(\varOmega )$ given by

18 $$\begin{aligned} J'(u) = - \lambda (0,\cdot ), \end{aligned}$$

where $\lambda$ is defined by (15), complemented with (16), (17). Even more, there exists a constant *d* such that

19 $$\begin{aligned} \left| J(v) - J(u) - \langle J'(u), v-u \rangle \right| \le d \Vert v- u \Vert ^2_{L_2(\varOmega )} \quad \forall \, u,\, v \in \mathcal{U}, \end{aligned}$$

where $\langle \cdot , \cdot \rangle$ is the scalar product in $L_2(\varOmega )$.

The proof of this proposition is given in Appendix 1.

### Implementation of the gradient projection method

Below we briefly describe, first at a conceptual level, our implementation of the gradient projection method for finding an “approximate” solution of problem (13), (6)–(10). Given $u_k \in \mathcal{U}$ at iteration *k* we define the next iteration as

$$\begin{aligned} u_{k+1} := \text{ pr }_\mathcal{U}(u_k + \rho _k J'(u_k)), \end{aligned}$$

where $\text{ pr }_\mathcal{U}$ is the projection operator on the set $\mathcal{U}$ with respect to the $L_2$-norm, and $\rho _k > 0$ is a step along the gradient $J'(u_k)$. (Notice that the projection in $L_2$ exists and is unique.) Although there are various reasonable ways of choosing the step size $\rho _k > 0$, we formulate a convergence result where the choice of $\rho _k$ is (to some extend) flexible.

#### Proposition 2

Let $d > 0$ be the number in Proposition 1 and let the real numbers $\rho _0 > 0$ and $\varepsilon > 0$ satisfy the inequality $\varepsilon \rho _0 < 1 - d \rho _0$. Then for any choice of $\rho _k \in [\rho _0, 1/(d + \varepsilon )]$ the sequence $\{u_k\}$ generated by the gradient projection method starting from an arbitrary $u_0 \in \mathcal{U}$ satisfies

20 $$\begin{aligned} \lim _k \sup _{u \in \mathcal{U}} \langle J'(u_k), u - u_k \rangle = 0. \end{aligned}$$

This statement of the proposition is known in principle, but due to the infinite dimensionality and the specificity in Proposition 1, which does not claim Fréchet differentiability in $L_2$, we present the proof (repeating usual arguments) in Appendix 2.

We point out that (20) represents an approximate version of the necessary optimality condition that a solution $u^*$ has to satisfy: $\langle J'(u^*), u - u^* \rangle \le 0$, $u \in \mathcal{U}$. Stronger convergence results can hardly be obtained in our infinite dimensional setting without additional assumptions of convexity and weak upper semi-continuity of *J*, which do not need to hold for our problem.

In the numerical implementation of the above conceptual method we pass to a finite-dimensional space by introducing meshes $\{\omega _i\}_{i=1}^M$ and $\{t_j\}_{j=0}^N$ in $\varOmega$ and [0, *T*], respectively. Then the systems (6)–(8) and (15)–(17) are replaced with discretizations obtained by application of a second order Runge–Kutta scheme (the Heun scheme) for the differential equations and the trapezoidal quadrature formula for integration over $\varOmega$. The approximation of the constraining set $\mathcal{U}$ has the polyhedral form

$$\begin{aligned} \mathcal{U}_M := \left\{ (u^1, \ldots , u^M) \in \mathbf{R}^{m\times M} : \,\varphi _0(\omega _i) \le u^i \le \varphi _1(\omega _i), \; \sum _{i=1}^{M} \alpha _i u^i = c \right\} , \end{aligned}$$

where $\alpha _i$ are the coefficients of the quadrature formula. In this way we obtain an approximating mathematical programming problem. The relation between the solution(s) of the obtained discretization problem and the solution(s) of the original problem (13), (6)–(10) is a subject of a separate investigation, similarly as in the recent paper (Veliov 2015), considering essentially the same (even more general) system, but in different class of controls.

Solving the discretized problem by the gradient projection method (with the gradient calculated by using the discretization of the adjoint equation) involves projection on the set $\mathcal{U}_M$. (Observe that $\mathcal{U}_M$ is non-empty for a sufficiently dense mesh $\{\omega _i\}_{i=1}^M$ due to Assumption (ii).) There is a huge literature and available software for this kind of projection problems, for both see e.g. Hager and Zhang (2015) and the references therein. For details about the implementation of the gradient projection method (including the choice of the step length $\rho$) see e.g. (Polak 1971, Chapter 4).

We mention that the known convergence results for the gradient projection method applied to the discrete problem are of the same kind as Proposition 2: claiming convergence to a “critical point”. In our numerical analysis we run the optimization solver for various initial guesses $u_0$. In the experiments with SI and SIR models (see the next section) we have never encountered convergence to a local (and non-global) maximizer.

We remind that to obtain a good approximation $\mathcal{E}_L(\tau )$ of the minimal convex set-membership estimation $\text{ pr }_L (\text{ co }\,\mathcal{R}(\tau ))$ for a given $\tau$ it is necessary to solve problem (13), (6)–(10) for many unit vectors *l* in the space of interest, *L*. Even more, in order to predict the evolution of state *y*(*t*) by means of the estimation $\mathcal{E}_L(t)$ we need to do this for a number of time instances $\tau$. Naturally, the obtained (approximate) maximizer *u* for given $\tau$ and *l* can be used as an initial guess for neighbouring instances $\tau$ and vectors *l*, which makes the overall estimation procedure tractable on a commercial PC.

## Numerical analysis

In this section we present numerical results and analysis of versions of SI and SIR heterogeneous models.

### SI-model without population growth

Here we deal with the system (1), (2) with $\kappa =0$, that is, the disease-free population has zero growth rate. We consider this special case for the following reason: the asymptotics of the minimal set-membership estimation $\mathcal{R}(t)$, $t \rightarrow +\infty$, can be determined in an alternative way, and can be compared with the estimation $\mathcal{E}(t)$ obtained by the approach in the present paper. This is a test for the performance of the set-estimation techniques. Let us briefly describe this alternative way.

From (1) it is apparent that $S(t,\omega )$ is monotonically decreasing and positive, and thus convergent. This easily implies that $\dot{S}(t,\omega )\rightarrow 0$. Since $\dot{I}(t,\omega )=-\dot{S}(t,\omega )-\gamma I(t,\omega )$, we obtain in a standard way that $I(t,\omega )$ converges to 0, provided that $\gamma > 0$. Thus also $I(t) \rightarrow 0$. In our paper (Veliov and Widder 2015) it is shown how to determine the asymptotics of *S*(*t*) for given initial data $(S(0,\cdot ), I(0,\cdot )) = (u_1, u_2)$. There, it is assumed that $p(\omega )=q(\omega ) > 0$, $\sigma (\omega ) = \sigma > 0$ is constant, and the set of those $\omega \in \varOmega$, for which $S(0,\omega )>0$ and $\gamma >\sigma p(\omega )$, has positive measure. Then Veliov and Widder (2015, Section 4.2) claims that

21 $$\begin{aligned} \begin{aligned} \lim _{t \rightarrow +\infty } S(t) := S^*(u_1,u_2) =\int _{\varOmega } e^{-\sigma F^*p(\omega )}u_1(\omega ) \text{ d }\omega , \end{aligned} \end{aligned}$$

where $F^*$ is the unique positive solution of the equation

$$\begin{aligned} \int _{\varOmega } p(\omega )e^{F^*(\gamma -\sigma p(\omega ))}u_1(\omega ) \text{ d }\omega = \int _{\varOmega } p(\omega )(u_1(\omega )+u_2(\omega )) \text{ d }\omega . \end{aligned}$$

Hence,

22 $$\begin{aligned} \lim _{t \rightarrow +\infty } \mathcal{R}(t) = \left[ \min _{(u_1,u_2) \in \mathcal{U}} S^*(u_1,u_2), \, \max _{(u_1,u_2) \in \mathcal{U}} S^*(u_1,u_2) \right] \times 0. \end{aligned}$$

Solving the two optimization problems involved in the last formula again requires a numerical algorithm, but now we deal with a completely static problems (differential equations are not involved). Again a gradient projection method is applied, since the Fréchet derivative of $S^*$ can be analytically represented. We skip the details of this procedure.

**Fig. 1:**
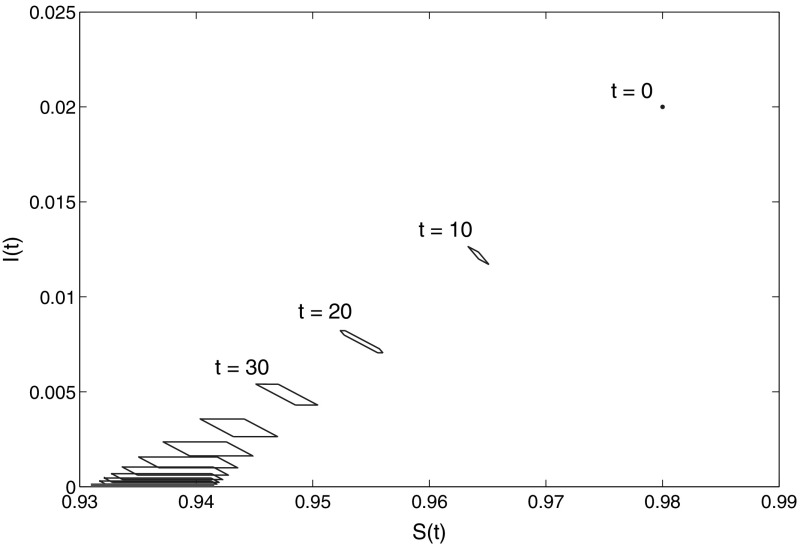
Set-membership estimates of system (1) with $\kappa =0$ for various *t*. The *thick line* at the *bottom left* is the exact set-estimation at infinity, $\mathcal{R}(+\infty )$, calculated as in (22). For $t<\infty$ the estimation $\mathcal{E}(t)$ is calculated by using 20 equidistant unit vectors $l \in \mathbf{R}^2$

**Fig. 2:**
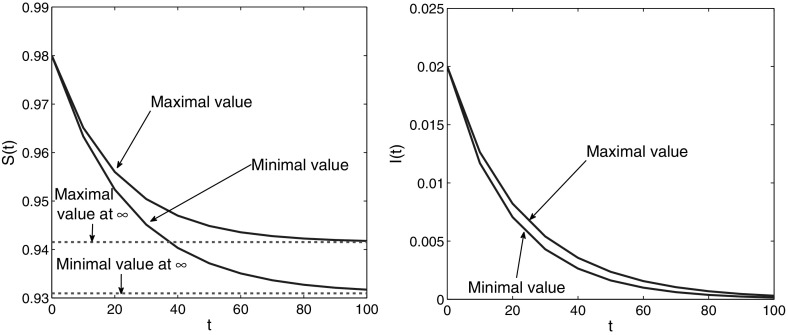
Estimates for the maximal and minimal value of *S*(*t*) and *I*(*t*). For large *t* these values for *S*(*t*) converge to the maximal and minimal value of $S^*$. For *I*(*t*) they converge to 0

The essence of the above paragraph is that now we have two different methods for approximation of the limit of $\mathcal{R}(t)$: by the way mentioned just above, and by using the general technique presented in this paper for approximating $\mathcal{R}(t)$, applied for large *t*. The comparison is clearly seen in Figs. 1 and 2, obtained for the data specifications described below.

The initial size of the population is normalized to one: $S(0)+I(0)=1$. Moreover, $\varOmega = [0,1]$, $\delta =0.15$ and $\sigma =0.1$. The weight functions $p(\omega )$ and $q(\omega )$ are linear: $p(\omega )=q(\omega )=0.5+\omega$ (the constant term means that all individuals have risky contacts). In order to define the lower and the upper bounds $\varphi _0(\cdot )$ and $\varphi _1(\cdot )$ of $u(\cdot )$ we assume that the initial distribution of trait $\omega$ among the susceptible and infected populations is close to a normal distribution $\varphi (\cdot )$ (called further “base distribution”) with mean 0.5 and variance 0.3 truncated to the unit interval and normalised there. More precisely, its deviation from $\varphi$ is at most 20 %. This leads to bounds

23 $$\begin{aligned} u_1(\omega ) \in \left[ \frac{4}{5}S(0) \varphi (\omega ),\frac{6}{5}S(0) \varphi (\omega )\right] , \qquad u_2(\omega ) \in \left[ \frac{4}{5}I(0) \varphi (\omega ), \frac{6}{5}I(0) \varphi (\omega )\right] . \end{aligned}$$

Figure 1 shows the evolution of the estimation $\mathcal{E}(t)$ obtained by using 20 equidistant unit vectors $l \in \mathbf{R}^2$. It converges to the limit set $\mathcal{R}(+\infty )$ calculated as in (22). Thus the two different ways to approximate the limit set-estimation are consistent with each other. This can be seen even better in Fig. 2 (left plot), where the dotted lines represent the interval in the right-hand side of (22), while the solid lines represent the evolution of $\text{ pr }_S (\mathcal{E}(t))$. The convergence of $\text{ pr }_I (\mathcal{E}(t))$ to zero is seen on the right plot in Fig. 2 (right plot).

### SI-model with population growth

**Fig. 3:**
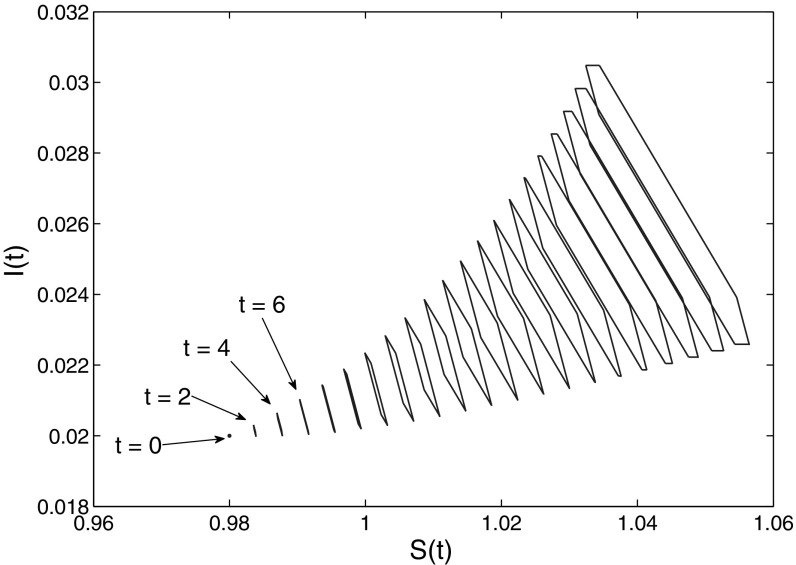
Set-membership estimates of system (1), (2) for various *t*, obtained by using 20 equidistant unit vectors $l \in \mathbf{R}^2$

We continue to deal with the system (1), (2), but now consider a growing population. We set $\delta =0.1$, $\sigma =0.105$, and $\kappa =0.004$. Figure 3 shows the set-membership estimation $\mathcal{E}(t)$ of the system (1) at $t = 2, 4, \dots , 40$, obtained by using 20 equidistant unit vectors $l \in \mathbf{R}^2$.

**Fig. 4:**
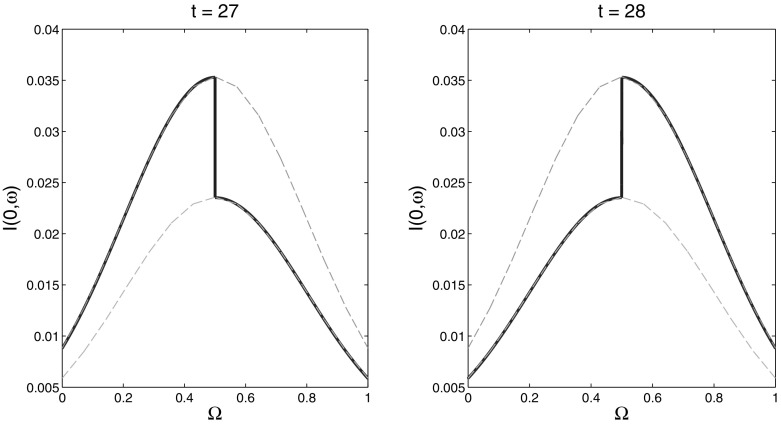
The *solid lines* show the component $u_2(\cdot )$ of the extreme data for direction $l=(\sin (1.4\pi ),\cos (1.4\pi ))$ and two different values of *t*. The *dashed lines* show the lower and upper bounds $\varphi _0^2(\omega )$ and $\varphi _1^2(\omega )$

We remind that obtaining a set-estimation $$\mathcal{E}(t)$$ requires solving the auxiliary problem (13) for various unit vectors *l* (in the present model case $$l \in \mathbf{R}^2$$). A feasible *u* that solves this problem is called extremal realization of the uncertainty in the initial data, or merely *extremal*, in direction *l*. A comprehensive analysis of the structure of the extremal *u* is a complicated task, seemingly not tractable, in general, although it may give useful information about “worst case” realizations of the uncertainty. Our numerical experiments with the SI model in Sect. 5.1 give evidence that the extremal *u* has a bang-bang structure. More precisely, for an extremal *u* there exists a subset $$A\subset \varOmega $$ such that $$u(\omega )=\varphi _1(\omega )$$ for $$\omega \in A$$ and $$u(\omega )=\varphi _0(\omega )$$ for $$\omega \in \varOmega \backslash A$$. Of course, the set *A* depends on *u*, hence on the estimation time *t* and the direction *l*. In the experiments with the present SI-model the set *A* always consists of a single interval. Figure 4 presents the extremal initial data $$u_2(\omega ) = I(0,\omega )$$ for $$l=(\sin (1.4\pi ),\cos (1.4\pi ))$$ and various values of *t*. For $$t=1,\dots ,27$$ the set *A* stays the same, $$A = [0,0.5)$$, and the corresponding $$u_2$$ is depicted on the left plot of Fig. 4. For $$t=28,\dots ,40$$ we obtain $$A = (0.5,1]$$ and the corresponding $$u_2$$ is depicted on the right plot of Fig. 4. Thus the structure of the extremal data may abruptly change when the estimation time changes.

In the rest of this subsection we investigate the effect of intervention (prevention) polices implemented prior to or around the outburst of the disease at $t=0$. Such a policy may influence either the individual susceptibility, $\sigma (\omega )$, (say, by vaccination) or the individual contact rate, $p(\omega )$ (by educational or other prevention programs). Assuming that the resource for intervention is limited, the question arises how to allocate it among individuals, regarding their *h*-state $\omega$. As mentioned in Sect. 2, exact information about the *h*-state of individuals is not available, therefore a complex intervention policy that targets specific sections of the population with particular *h*-states cannot be enforced in practice. However, it may be feasible to identify groups of high-level and groups of low-level risk.

In view of the above, we consider two scenarios: applying the intervention to high risk individuals (here we mean those with high values of $p(\omega )$) or applying it to low risk individuals (i.e. those with low values of $p(\omega )$, respectively). Even though this way of modelling of interventions is crude, it qualitatively answers the question which part of the population (high risk or low risk individuals) should be mainly targeted. As we see below, the answer is not evident.

To be specific, we assume that for a third of the population we can decrease susceptibility $\sigma (\omega )$ or the contact rate $p(\omega )$ by 50 %. The question is, what will the effect of the intervention be if it is applied to the one third of the population at higher risk versus the same fraction of the population at low risk. The effect of intervention is measured by the set-membership estimation of the prevalence. Let us clarify the last notion. If we have obtained a set-estimation $\mathcal{E}(t)$ for (*S*(*t*), *I*(*t*)), then the corresponding set-estimation for the prevalence $I(t)/(S(t)+I(t))$ is the interval

$$\begin{aligned} \mathcal{E}_p(t) := \left[ \min _{(s,i) \in \mathcal{E}(t)} \frac{i}{s+i}, \, \max _{(s,i) \in \mathcal{E}(t)} \frac{i}{s+i} \right] . \end{aligned}$$

**Fig. 5:**
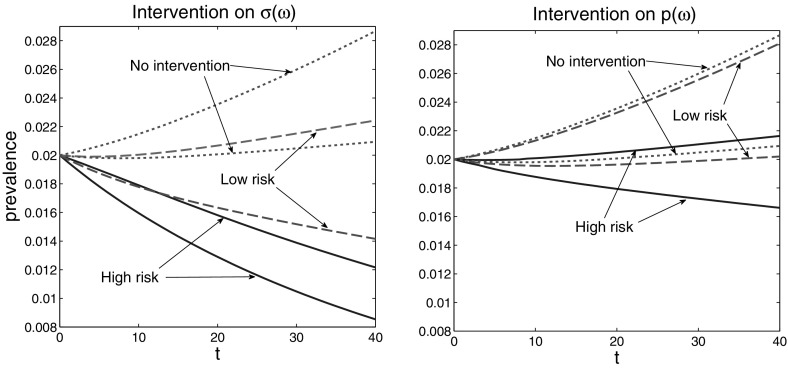
Set-estimations of the prevalence, $\mathcal{E}_p(t)$, in case of intervention affecting $\sigma (\omega )$ (*left plot*) and $p(\omega )$ (*right plot*). The *dotted lines* represent $\mathcal{E}_p(t)$ in the case of no intervention, the *dashed lines* represent $\mathcal{E}_p(t)$ in the case of intervention applied to low risk individuals, and the *solid lines*—to high risk individuals

In Fig. 5 we show the progress of the set-estimation of the prevalence in three scenarios: no intervention (the dotted lines), intervention applied to low risk individuals (dashed lines), and intervention applied to high risk individuals (solid lines). On the left plot the intervention decreases the susceptibility $\sigma (\omega )$ of the treated individuals, while on the right plot—the contact rates, $p(\omega )$. Comparing the figures we see that in both cases interventions are productive and that the intervention applied to high risk individuals is significantly more efficient.

**Fig. 6:**
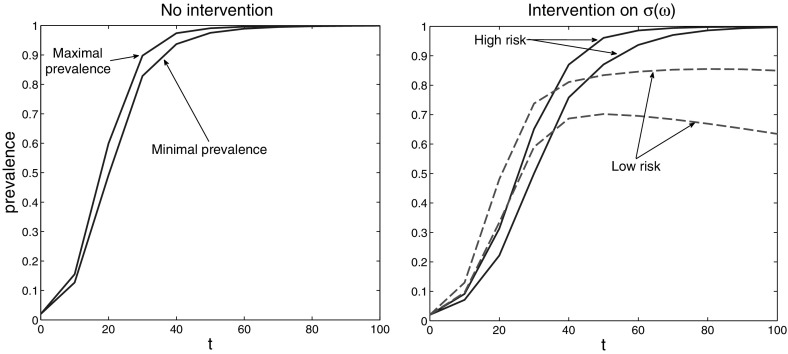
The *left plot* shows the evolution of the set-estimation $\mathcal{E}_p(t)$ of the prevalence in case of no intervention: the whole population becomes (asymptotically) infected. The *right plot* shows $\mathcal{E}_p(t)$ with the intervention applied to the low risk (*dashed lines*) and high risk (*solid lines*) individuals. The intervention targeting the low risk individuals is now significantly more efficient and, in particular, prevents extinction

However, it is not always the case that intervention is more efficient if applied to the individuals with highest risk. To show this we consider the above SI model with only the value of $\sigma (\omega )$ increased from 0.105 to 0.3, i.e. we assume higher susceptibility. On the left plot of Fig. 6 we see that the prevalence approaches value 1 (and actually the population becomes extinct, asymptotically). We consider again an intervention that reduces $\sigma (\omega )$ by 50 %. On the right plot of Fig. 6 we see the result of this intervention when applied to the high risk and to low risk individuals, respectively. Again both interventions yield an improvement. However, now the intervention targeting the low risk individuals is more efficient and prevents extinction.

### Comparison with other distributions of the initial data

In Sect. 5.2 we assume that the base information, $\varphi$, about the initial data is a normal distribution truncated to the interval [0, 1] and normalised so that $\int _0^1\varphi (\omega ) \text{ d }\omega =1$. Now, we make a comparison with results for other choices of $\varphi$. We consider a uniform distribution, i.e. $\varphi (\omega )=1$, and two distributions skewed to either side of the interval [0, 1], namely, $\varphi (\omega )=C_1(e^{\omega }-1)$ and $\varphi (\omega )=C_2(e^{-\omega +1}-1)$, where $C_1$ and $C_2$ are appropriate normalising constants. We will refer to the first of these two functions as ”right-hand exponential” and to the second one as ”left-hand exponential”. As before (see (23)), a 20 % deviation of the real data from the base ones is considered as possible.

**Fig. 7:**
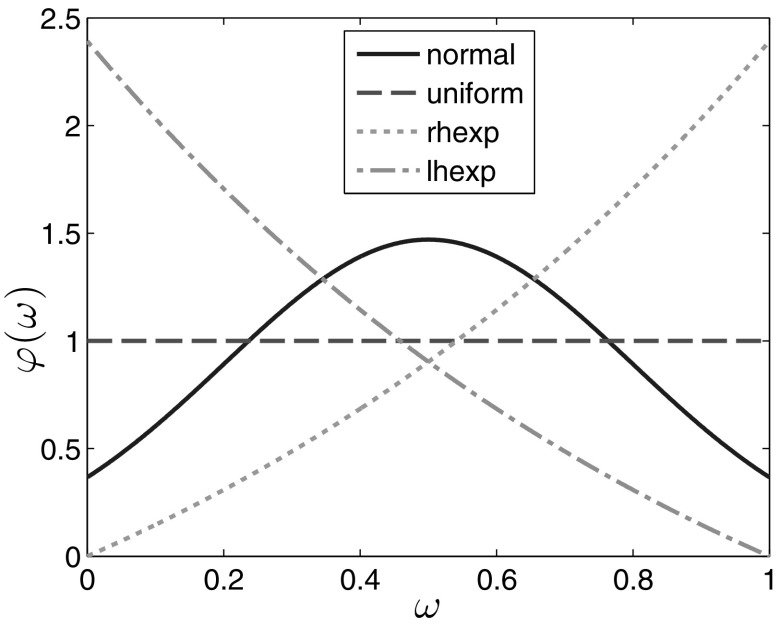
The four base distributions $\varphi$ considered in Sect. 5.3

The different choices of $\varphi$, including the normal distribution used before, are shown in Fig. 7 .

**Fig. 8:**
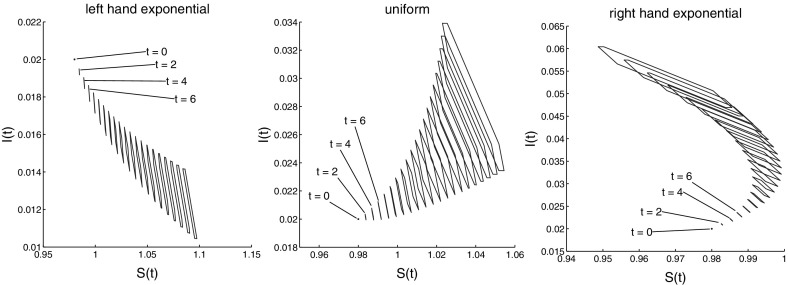
Set-estimations of (*S*, *I*) for various time instances for left-hand exponential, uniform, and right-hand exponential base distribution $\varphi$

**Fig. 9:**
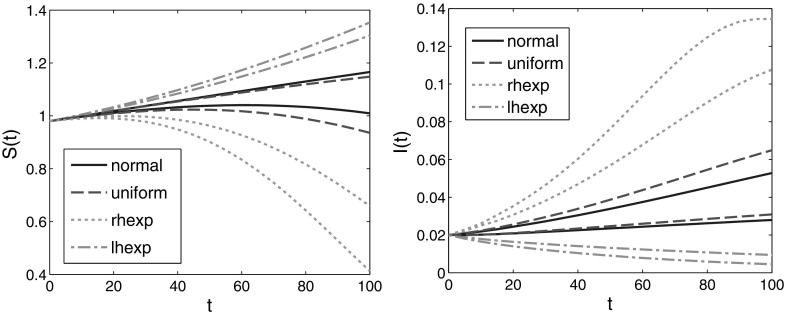
Separate set-estimations of *S* and *I* for various base distributions $\varphi$

Figure 8 presents the set-membership estimations corresponding to uniform, right-shifted and left-shifted base distributions. It should be viewed in parallel with Fig. 3, which corresponds to a normal base distribution $\varphi$. Figure 9 shows the separate estimates for *S*(*t*) and *I*(*t*) on a long time-horizon.

The results correspond to the common sense. The more to the right is shifted the base distribution $\varphi$ (meaning that more individuals have higher contact rates $p(\omega ) = 0.5 + \omega$), the more are the set-estimates shifted to higher number of infected individuals. It is interesting to observe that the size of the set-estimations increases along the line “uniform” $\rightarrow$ “normal” $\rightarrow$ “left-hand exponential” $\rightarrow$ “right-hand exponential” distribution of the base data $\varphi$. This is apparently related to differences in the stability of the model (1) for different initial data.

### SIR-model

In this subsection we consider the following heterogeneous SIR model:

24 $$\begin{aligned} \dot{S}(t,\omega )= & {} \kappa N(\omega )-\sigma p(\omega )J(t)S(t,\omega )-\kappa S(t,\omega ),\quad S(0,\omega )=u_1(\omega )\nonumber \\ \dot{I}(t,\omega )= & {} \sigma p(\omega )J(t)S(t,\omega )-(\gamma +\kappa )I(t,\omega ),\quad I(0,\omega )=u_2(\omega )\nonumber \\ \dot{R}(t,\omega )= & {} \gamma I(t,\omega )-\kappa R(t,\omega ),\quad R(0,\omega )=u_3(\omega ), \end{aligned}$$

where

$$\begin{aligned} \begin{aligned} J(t)&=\int _\varOmega p(\omega )I(t,\omega ) \text{ d }\omega . \end{aligned} \end{aligned}$$

The new variable $R(t,\omega )$ represents the “number” of individuals who have recovered from the infection. Now the parameter $\gamma$ has to be interpreted as the recovery rate, and $\kappa$ denotes both the birth rate and the mortality rate. The function $N(\omega )=S(t,\omega )+I(t,\omega )+R(t,\omega )$ describes the total population and is constant in time, as can be seen by summing up all three equations in (24). Thus in this model the disease has no influence on the mortality of infected individuals. Furthermore, newborn individuals are assumed to be susceptible and reproduction is not affected by being infected or recovered. Notice that the denominator of the weighted prevalence (compare with the SI model (1)) is missing. The reason is, that here we assume (for a simplification that is actually not necessary) that the susceptible, the infected, and the recovered individuals of *h*-state $\omega$ have the same contact rate $p(\omega )$. Then the weighted prevalence is given by

25 $$\begin{aligned} \frac{J(t)}{\int _\varOmega p(\omega ) (S(t,\omega ) + I(t,\omega ) + R(t,\omega )) \text{ d }\omega } = \frac{J(t)}{\int _\varOmega p(\omega ) N(\omega ) \text{ d }\omega }. \end{aligned}$$

Normalizing the denominator in the rightmost expression in (25) to 1 we obtain the simplified model (24).

**Fig. 10:**
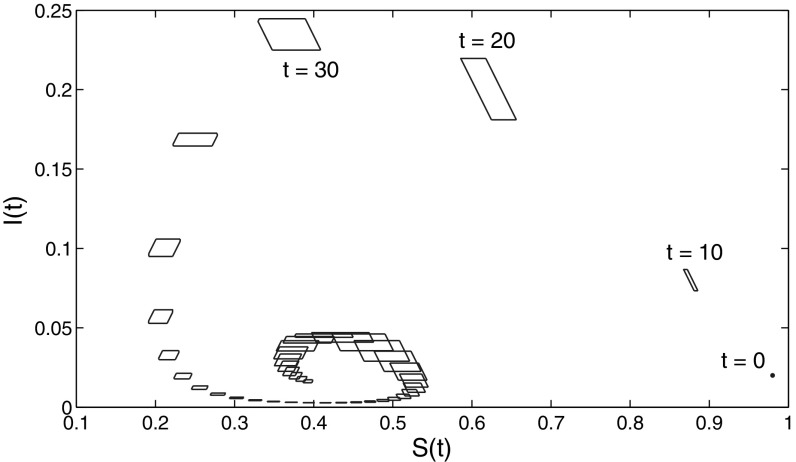
Set-membership estimates on the (*S*, *I*)-plane of system (24) for various times *t*, obtained by using 8 equidistant unit vectors $l \in \mathbf{R}^2\times 0$

In Fig. 10 we show the progress of the set-estimation of (*S*(*t*), *I*(*t*)) for $\sigma =0.25$, $\kappa =0.004$, $\gamma =0.1$, and $p(\omega )=0.5+\omega$. We assume that at $t=0$ there are no recovered individuals, i.e. $u_3(\omega )=0$, and the bounds on $u_1(\cdot )$ and $u_2(\cdot )$ are the same as in Sect. 5.1. We see that the set-estimation exhibits an oscillatory behaviour, in contrast with the SI-model. The size of the set-estimation varies with time, but remains reasonably small.

**Fig. 11:**
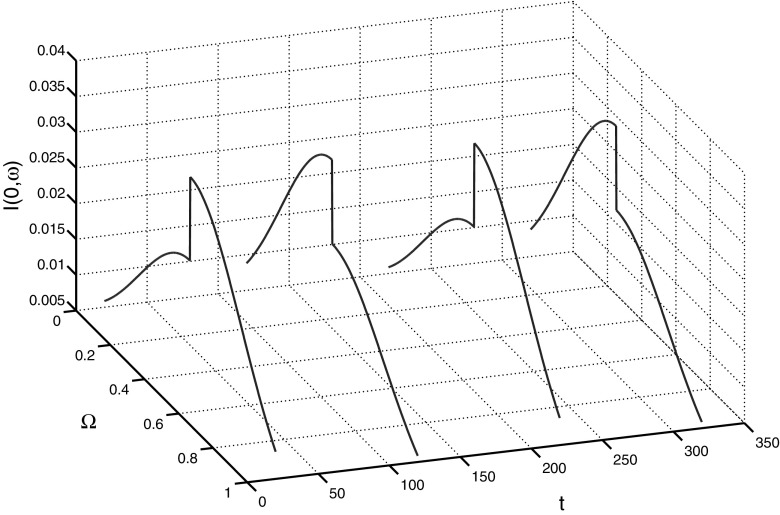
Extremal initial data $u_2(\omega ) = I(0,\omega )$ corresponding to $l= (0,1)$ and times $t=20,120,220,320$

It is interesting to mention that the structure of the extremal initial data $(u_1(\omega ),u_2(\omega ),0)$ (see Sect. 5.2) in the SIR-model is much more complicated than that in the SI-model. As seen in Fig. 11, for a given unit vector $l \in L := \mathbf{R}^2 \times 0$, the extremal initial data $u_2(\omega ) = I(0,\omega )$ may change its structure several times when the estimation time progresses: for time instances $t = 20$ and $t = 220$ the lower bound $\varphi _0^2(\omega )$ is active for small $\omega$ and the upper bound $\varphi _1^2(\omega )$ is active for large $\omega$, while for time instances $t = 120$ and $t = 320$ the opposite happens.

## Conclusions and perspectives

In this paper we demonstrate the tractability and applicability of the set-membership estimation approach for prediction of the evolution of infectious diseases in heterogeneous populations, using distributed differential models under uncertainty about the individual traits relevant for the disease. The available information is in the form of two-sided bounds for the distribution of the initial population along the space of heterogeneity (the *h*-states), possibly together with some aggregated data. Although the numerical illustrations of the developed estimation technique involve only SI and SIR heterogeneous models, the technique is applicable to more complex models, provided that the evolution of only 2 or 3 aggregated states (such as the total number of susceptible, infected, recovered, etc. individuals) have to be estimated.

However, the presented general model has the drawback that the individuals do not change their *h*-state (that is, their individual traits) over time. If the trait comprises the contact rate, this means that individuals keep their contact rate constant, independently of the evolution of the disease. Change of the contact rate may happen only if an individual becomes infected. This assumption is not realistic, and models and corresponding estimation techniques aimed to cope with variable individual traits are a subject of current work.

Another line of research is to involve in the presented model framework *dynamic* intervention policies (not only prevention prior to the outburst of the disease, as in Sect. 5.2 of the present paper). The uncertainty about the *h*-states of the population brings into consideration the problem to control the evolution of set-membership estimations by prevention or medication policies. This problem is profoundly investigated in other, mainly engineering, contexts (see the recent book Kurzhanski and Varaiya 2014 and the numerous references therein).

## Appendix 1: Proof of Proposition 1

Let $u(\cdot )$ be an admissible control and $\varDelta u(\cdot )$ be such that $u(\cdot ) + \varDelta u(\cdot )$ is admissible too. We shall denote by $x(\cdot ,\cdot ) + \varDelta x(\cdot ,\cdot )$, and by $y(\cdot ) + \varDelta y(\cdot )$ the state variables corresponding to $u(\cdot ) + \varDelta u(\cdot )$ and by *J*(*u*) the functional $\left\langle l,y[u](\tau ) \right\rangle$ (we remind that *l* is a given nonzero vector from $\mathbf{R}^n$). We also introduce the notational convention to skip the arguments *x* and *y*. For instance $f(t,\omega ) := f(t,\omega ,x(t,\omega ),y(t))$, $g'_x(t,\omega ) := g'_x(t,\omega ,x(t,\omega ))$, etc.

Let us define the number *r* as

$$\begin{aligned} r = J(u + \varDelta u)-J(u)+\int _{\varOmega }\langle \lambda (0,\omega ),\varDelta u(\omega )\rangle \text{ d }\omega \end{aligned}$$

We will show that *r* fulfills $|r| \le C \Vert \varDelta (\cdot )\Vert _{L_2(\varOmega )}^2$ for some positive constant *C*, which together with the remark at the end of the proof implies the claim of the proposition. First we give a more explicit representation of *r*.

### Lemma 2

The remainder *r* fulfils

$$\begin{aligned} r= & {} - \int _0^{\tau } \int _\varOmega \left\langle \lambda (t,\omega ), f'_y(t,\omega ) \int _\varOmega [ g'_x(t,\omega ',{\tilde{x}}(t,\omega '))\right. \\&\left. -g'_x(t,\omega ')] \varDelta x(t,\omega ') \text{ d }\omega '\right\rangle \text{ d }\omega \text{ d }t \\&+ \int _0^{\tau } \int _\varOmega \left\langle \lambda (t,\omega ), [f'_x(t,\omega ,{\bar{x}}(t,\omega ), {\bar{y}}(t)) - f'_x(t,\omega )] \varDelta x(t,\omega )\right\rangle \text{ d }\omega \text{ d }t \\&+ \int _0^{\tau } \int _\varOmega \left\langle \lambda (t,\omega ), [f'_y(t,\omega ,{\bar{x}}(t,\omega ), {\bar{y}}(t)) - f'_y(t,\omega )] \varDelta y(t)\right\rangle \text{ d }\omega \text{ d }t\\&+ \int _\varOmega \left\langle l, \left[ g'_x(\tau ,\omega ,{\tilde{x}}(\tau ,\omega )) - g'_x(\tau ,\omega ) \right] \varDelta x(\tau ,\omega ) \right\rangle \text{ d }\omega \end{aligned}$$

where ${\bar{x}}(t,\omega )$ and ${\tilde{x}}(t,\omega )$ lie between $x(t,\omega )$ and $x(t,\omega ) + \varDelta x(t,\omega )$ for $(t,\omega ) \in D$ and ${\bar{y}}(t)$ lies between *y*(*t*) and $y(t) + \varDelta y(t)$ for $t \in [0,\tau ]$.

### Proof

Using (6) in the last term in the third row below, we obtain

26 $$\begin{aligned}&\int _0^{\tau } \int _\varOmega \left\langle {\dot{\lambda }}(t,\omega ), \varDelta x(t,\omega )\right\rangle \text{ d }\omega \text{ d }t\nonumber \\&\quad =\int _0^{\tau } \frac{d}{dt} \left( \int _\varOmega \left\langle \lambda (t,\omega ), \varDelta x(t,\omega )\right\rangle \text{ d }\omega \right) \text{ d }t\nonumber \\&\qquad - \int _0^{\tau } \int _\varOmega \left\langle \lambda (t,\omega ), {\dot{\varDelta }} x(t,\omega )\right\rangle \text{ d }\omega \text{ d }t \nonumber \\&\quad = \int _\varOmega \left\langle \lambda (\tau ,\omega ), \varDelta x(\tau ,\omega )\right\rangle \text{ d }\omega - \int _\varOmega \left\langle \lambda (0,\omega ), \varDelta x(0,\omega )\right\rangle \text{ d }\omega \nonumber \\&\qquad - \int _0^{\tau } \int _\varOmega \left\langle \lambda (t,\omega ), {\dot{\varDelta }} x(t,\omega )\right\rangle \text{ d }\omega \text{ d }t \nonumber \\&\quad = \int _\varOmega \left\langle \lambda (\tau ,\omega ), \varDelta x(\tau ,\omega )\right\rangle \text{ d }\omega - \int _\varOmega \left\langle \lambda (0,\omega ), \varDelta x(0,\omega )\right\rangle \text{ d }\omega \nonumber \\&\qquad - \int _0^{\tau } \int _\varOmega \left\langle \lambda (t,\omega ), f'_x(t,\omega ) \varDelta x(t,\omega ) + f'_y(t,\omega ) \varDelta y(t)\right\rangle \text{ d }\omega \text{ d }t \nonumber \\&\qquad + \int _0^{\tau } \int _\varOmega \left\langle \lambda (t,\omega ), [f'_x(t,\omega ,{\bar{x}}(t,\omega ), {\bar{y}}(t)) - f'_x(t,\omega )] \varDelta x(t,\omega )\right\rangle \text{ d }\omega \text{ d }t \nonumber \\&\qquad + \int _0^{\tau } \int _\varOmega \left\langle \lambda (t,\omega ), [f'_y(t,\omega ,{\bar{x}}(t,\omega ), {\bar{y}}(t)) - f'_y(t,\omega )] \varDelta y(t)\right\rangle \text{ d }\omega \text{ d }t, \end{aligned}$$

where ${\bar{x}}(t,\omega )$ is between $x(t,\omega )$ and $x(t,\omega ) + \varDelta x(t,\omega )$ for $(t,\omega ) \in D$ and ${\bar{y}}(t)$ is between *y*(*t*) and $y(t) + \varDelta y(t)$ for $t \in [0,\tau ]$. It is standard to prove that ${\bar{x}}$ and ${\bar{y}}$ may be chosen measurable. Also, for each $t \in [0,\tau ]$ we have

27 $$\begin{aligned} \varDelta y(t)= & {} \int _\varOmega [g(t,\omega ,x(t,\omega ) + \varDelta x(t,\omega )) - g(t,\omega )] \text{ d }\omega \nonumber \\= & {} \int _\varOmega g'_x(t,\omega ) \varDelta x(t,\omega ) \text{ d }\omega + \int _\varOmega [g'_x(t,\omega ,{\tilde{x}}(t,\omega ))\nonumber \\&- g'_x(t,\omega ))] \varDelta x(t,\omega ) \text{ d }\omega \end{aligned}$$

where ${\tilde{x}}(t,\omega )$ is between $x(t,\omega )$ and $x(t,\omega ) + \varDelta x(t,\omega )$ for $(t,\omega ) \in D$, and is also measurable. Substituting $\varDelta y(t)$ from (27) into the fifth row of (26), we obtain

28 $$\begin{aligned}&\int _0^{\tau } \int _\varOmega \left\langle {\dot{\lambda }}(t,\omega ), \varDelta x(t,\omega )\right\rangle \text{ d }\omega \text{ d }t\nonumber \\&\quad =\int _\varOmega \left\langle \lambda (\tau ,\omega ), \varDelta x(\tau ,\omega )\right\rangle \text{ d }\omega \nonumber \\&\qquad - \int _\varOmega \left\langle \lambda (0,\omega ), \varDelta x(0,\omega )\right\rangle \text{ d }\omega \nonumber \\&\qquad - \int _0^{\tau } \int _\varOmega \left\langle \lambda (t,\omega ), f'_x(t,\omega ) \varDelta x(t,\omega )\right. \nonumber \\&\qquad \left. + f'_y(t,\omega ) \int _\varOmega g'_x(t,\omega ') \varDelta x(t,\omega ') \text{ d }\omega ' \right\rangle \text{ d }\omega \text{ d }t \nonumber \\&\qquad - \int _0^{\tau } \int _\varOmega \left\langle \lambda (t,\omega ), f'_y(t,\omega ) \int _\varOmega [g'_x(t,\omega ' ,{\tilde{x}}(t,\omega ' ))\right. \nonumber \\&\qquad \left. - g'_x(t,\omega ' ))] \varDelta x(t,\omega ' ) \text{ d }\omega ' \right\rangle \text{ d }\omega \text{ d }t \nonumber \\&\qquad + \int _0^{\tau } \int _\varOmega \left\langle \lambda (t,\omega ), [f'_x(t,\omega ,{\bar{x}}(t,\omega ), {\bar{y}}(t)) - f'_x(t,\omega )] \varDelta x(t,\omega )\right\rangle \text{ d }\omega \text{ d }t \nonumber \\&\qquad + \int _0^{\tau } \int _\varOmega \left\langle \lambda (t,\omega ), [f'_y(t,\omega ,{\bar{x}}(t,\omega ), {\bar{y}}(t)) - f'_y(t,\omega )] \varDelta y(t)\right\rangle \text{ d }\omega \text{ d }t. \end{aligned}$$

Denote the last three terms in (28) by ${\tilde{r}}$. In the first three terms we substitute ${\dot{\lambda }}(t,\omega )$ and $\varDelta x(0,\omega )$ from (15) and (7), respectively. Using also (17), we rewrite the above equality as

29 $$\begin{aligned} 0 = \int _\varOmega \left\langle \lambda (\tau ,\omega ), \varDelta x(\tau ,\omega ) \right\rangle \text{ d }\omega - \int _\varOmega \left\langle \lambda (0,\omega ), \varDelta u(\omega ) \right\rangle \text{ d }\omega + {\tilde{r}}. \end{aligned}$$

On the other hand we have

30 $$\begin{aligned} J(u + \varDelta u) - J(u)= & {} \left\langle l,y(\tau ) + \varDelta y(\tau )\right\rangle - \left\langle l,y(\tau ) \right\rangle \nonumber \\= & {} \left\langle l,\int _\varOmega [ g(\tau ,\omega ,x(\tau ,\omega ) + \varDelta x(\tau ,\omega )) - g(\tau ,\omega )] \text{ d }\omega \right\rangle \nonumber \\= & {} \left\langle l,\int _\varOmega g'_x(\tau ,\omega ) \varDelta x(\tau ,\omega ) \text{ d }\omega \right\rangle \nonumber \\&+ \left\langle l,\int _\varOmega [g'_x(\tau ,\omega ,{\tilde{x}}(\tau ,\omega )) - g'_x(\tau ,\omega )] \varDelta x(\tau ,\omega ) \text{ d }\omega \right\rangle .\qquad \qquad \end{aligned}$$

Adding (29) to this equality and taking into account (16) we obtain

$$\begin{aligned} J(u+ \varDelta u) - J(u)= & {} - \int _\varOmega \left\langle \lambda (0,\omega ), \varDelta u(\omega )\right\rangle \text{ d }\omega +{\tilde{r}} + \int _\varOmega \left\langle l, [g'_x(\tau ,\omega ,{\tilde{x}}(\tau ,\omega ))\right. \\&\left. - g'_x(\tau ,\omega )] \varDelta x(\tau ,\omega ) \right\rangle \text{ d }\omega , \end{aligned}$$

which, in view of the definition of ${\tilde{r}}$, implies the claim of the lemma. $\square$

We next estimate the four terms in the remainder *r*.

### Lemma 3

The remainder *r* satisfies the estimate

31 $$\begin{aligned} |r| \le C \Vert \varDelta u(\cdot ) \Vert ^2_{L_{2}(\varOmega )}, \end{aligned}$$

where *C* is a constant.

### Proof

As in (27) we have

$$\begin{aligned} \varDelta y(t)= & {} \int _{\varOmega } [g(t,\omega ,x(t,\omega )+\varDelta x(t,\omega )) - g(t,\omega )] \text{ d }\omega \\= & {} \int _\varOmega g'_x(t,\omega ,{\tilde{x}}(t,\omega )) \varDelta x(t,\omega ) \text{ d }\omega , \end{aligned}$$

for each $t \in [0,\tau ]$. Because of the local essential boundedness of $g'_x$, we obtain

32 $$\begin{aligned} |\varDelta y(t)| \le C_1 \int _\varOmega |\varDelta x(t,\omega )| \text{ d }\omega \end{aligned}$$

for each $t \in [0,\tau ]$, where $C_1$ is some positive constant (as are $C_2$, $C_3$, etc., below). Because of (11), as in (26) we obtain

$$\begin{aligned} \varDelta x(t,\omega )= & {} \varDelta u(\omega ) + \int _0^t [f'_x(s,\omega ,{\bar{x}}(s,\omega ), {\bar{y}}(s)) \varDelta x(s,\omega ) \\&+ f'_y(s,\omega ,{\bar{x}}(s,\omega ), {\bar{y}}(s)) \varDelta y(s)] \text{ d }s. \end{aligned}$$

for $(t,\omega ) \in [0,\tau ] \times \varOmega ' \subset D$ ($\varOmega '$ being of full Lebesgue measure in $\varOmega$). Hence, using (32) and the local essential boundedness of $f'_x$ and $f'_y$, we have

33 $$\begin{aligned} |\varDelta x(t,\omega )| \le |\varDelta u(\omega )| + C_2 \int _0^t \Big [ |\varDelta x(s,\omega )| + \int _\varOmega |\varDelta x(s,\omega )| \text{ d }\omega \Big ] \text{ d }s. \end{aligned}$$

for $(t,\omega ) \in [0,\tau ] \times \varOmega ' \subset D$ ($\varOmega '$ of full Lebesgue measure in $\varOmega$). Integrating the above inequality in $\omega$ over $\varOmega$ and changing the order of integration where necessary, we obtain that the function

$$\begin{aligned} \delta (t) \mathop {=}\limits ^{def}\int _\varOmega |\varDelta x(t,\omega )| \text{ d }\omega \end{aligned}$$

satisfies

$$\begin{aligned} \delta (t) \le \Vert \varDelta u(\cdot ) \Vert _{L_{_1}(\varOmega )} + C_2(1 + {{\mathrm{meas}}}\{\varOmega \}) \int _0^t \delta (s) \text{ d }s \end{aligned}$$

and the Grönwall inequality yields

34 $$\begin{aligned} \delta (t) \le C_3 \Vert \varDelta u(\cdot ) \Vert _{L_{_1}(\varOmega )} \end{aligned}$$

for all $t \in [0,\tau ]$. Substituting (34) into (33), we obtain

35 $$\begin{aligned} |\varDelta x(t,\omega )|\le & {} |\varDelta u(\omega )| + C_2 \int _0^t |\varDelta x(s,\omega )| \text{ d }s + C_2 \int _0^t \delta (s) \text{ d }s \nonumber \\\le & {} |\varDelta u(\omega )| + C_2 C_3 \tau \Vert \varDelta u(\cdot ) \Vert _{L_{_1}(\varOmega )} + C_2 \int _0^t |\varDelta x(s,\omega )| \text{ d }s \end{aligned}$$

for almost every $(t,\omega ) \in [0,\tau ] \times \varOmega$. Using again the Grönwall inequality, we obtain

36 $$\begin{aligned} |\varDelta x(t,\omega )| \le C_4 \left( | \varDelta u(\omega ) | + \Vert \varDelta u(\cdot ) \Vert _{L_{_{1}}(\varOmega )}\right) . \end{aligned}$$

From here

37 $$\begin{aligned} \Vert \varDelta x(t,\cdot )\Vert _{L_1(\varOmega )} \le C_4(1 + \mathrm{meas}(\varOmega )) \, \Vert \varDelta u(\cdot ) \Vert _{L_{_{1}}(\varOmega )} \le C_5 \Vert \varDelta u(\cdot ) \Vert _{L_{_{2}}(\varOmega )}. \end{aligned}$$

Then using (32) we obtain that

38 $$\begin{aligned} |\varDelta y(t)| \le C_1 C_5 \Vert \varDelta u(\cdot ) \Vert _{L_{_{2}}(\varOmega )}. \end{aligned}$$

Using the estimations (36), (37) and (38), taking into account Assumptions (i) and (iii) and the boundedness of $\lambda$ (Lemma 1) we obtain that each of the terms in the definition of the remainder *r* can be estimated as in the statement of the lemma. $\square$

To finish the prof of Proposition 1 we observe that all constants that appear above can be chosen independent of *u* and $\varDelta u$, provided that both *u* and $u + \varDelta u$ belong to $\mathcal{U}$. This is due to the uniformity in Assumption (iii) and in Lemma 1.

## Appendix 2: Proof of Proposition 2

Since $u_{k+1}$ solves the minimization problem $\min _{u \in \mathcal{U}} \Vert u_k + \rho _k J'(u_k) - u\Vert ^2_{L_2(\varOmega )}$, we have (the first order optimality condition)

39 $$\begin{aligned} 2 \left\langle u_k + \rho _k J'(u_k) - u_{k+1}, u - u_{k+1} \right\rangle \le 0 \quad \text{ for } \text{ every } u \in \mathcal{U}. \end{aligned}$$

Applying this inequality for $u = u_k$ we obtain that

$$\begin{aligned} \rho _k \left\langle J'(u_k), u_{k+1} - u_k \right\rangle \ge \Vert u_{k+1} - u_k \Vert _{L_2(\varOmega )}^2. \end{aligned}$$

On the other hand, we obtain from Proposition 1 that

$$\begin{aligned} \begin{aligned} J(u_{k+1}) - J(u_k)&\ge \left\langle J'(u_k), u_{k+1} - u_k \right\rangle - d \Vert u_{k+1} - u_k\Vert ^2_{L_2(\varOmega )}\\&\ge \left( \frac{1}{\rho _k} - d \right) \Vert u_{k+1} - u_k\Vert ^2_{L_2(\varOmega )} \ge \varepsilon \Vert u_{k+1} - u_k\Vert ^2_{L_2(\varOmega )}. \end{aligned} \end{aligned}$$

Thus the sequence $\{J(u_k)\}$ is monotone increasing, and due to the uniform boundedness in Assumption (iii) this sequence is also bounded, hence convergent. Then the last inequality implies that the sequence $\varepsilon _k := \Vert u_{k+1} - u_k\Vert _{L_2(\varOmega )}$ converges to zero.

From (39) we obtain that for every $u \in \mathcal{U}$

$$\begin{aligned} \rho _k \left\langle J'(u_k), u - u_{k+1} \right\rangle \le \langle u_{k+1} - u_k, u - u_{k+1} \rangle . \end{aligned}$$

Hence

$$\begin{aligned} \rho _k \left\langle J'(u_k), u - u_{k} \right\rangle\le & {} \rho _k {\bar{c}} \left( \mathrm{meas}(\varOmega )\right) ^{\frac{1}{2}} \Vert u_{k+1} - u_k\Vert _{L_2(\varOmega )}\\&+ \Vert u_{k+1} - u_k\Vert _{L_2(\varOmega )} \Vert u - u_{k+1} \Vert _{L_2(\varOmega )}, \end{aligned}$$

where ${\bar{c}}$ is the constant from Lemma 1. From here we obtain

$$\begin{aligned} \left\langle J'(u_k), u - u_{k} \right\rangle \le \left( {\bar{c}} \left( \mathrm{meas}(\varOmega )\right) ^{\frac{1}{2}} + \frac{\mathrm{diam}(\mathcal{U})}{\rho _0} \right) \Vert u_{k+1} - u_k\Vert _{L_2(\varOmega )} = C \varepsilon _k, \end{aligned}$$

where *C* is the constant in the parentheses. The convergence of $\varepsilon _k$ to zero implies the claim of the proposition.
